# Multi-Omics Profiling of Individuals Sustaining Extreme Physical Stressors

**DOI:** 10.3390/life15091377

**Published:** 2025-09-01

**Authors:** Anurag Sakharkar, Robert Chen, Erik LeRoy, Theodore M. Nelson, Jacqueline Proszynski, JangKeun Kim, Jiwoon Park, Mohith Reddy Arikatla, Begum Mathyk, Christopher E. Mason

**Affiliations:** 1 Department of Physiology and Biophysics, Weill Cornell Medicine, New York, NY 10021, USA; anurag.skhrkr@gmail.com (A.S.); jgr9007@med.cornell.edu (R.C.); thn4005@med.cornell.edu (T.M.N.); jap7033@med.cornell.edu (J.P.); jak4013@med.cornell.edu (J.K.); jip2007@med.cornell.edu (J.P.); moa4020@med.cornell.edu (M.R.A.); 2 Department of Pathology and Laboratory Medicine, Weill Cornell Medicine, New York, NY 10021, USA; 3 Association of Spaceflight Professionals, Tampa, FL 33607, USA; erik.leroy@spaceflightprofessionals.org; 4 NYU Grossman School of Medicine, New York, NY 10016, USA; 5 The HRH Prince Alwaleed Bin Talal Bin Abdulaziz Alsaud Institute for Computational Biomedicine, Weill Cornell Medicine, New York, NY 10021, USA; 6 Department of Obstetrics and Gynecology, University of South Florida Morsani College of Medicine, Tampa, FL 33602, USA; abegum@usf.edu; 7 Department of Obstetrics, Gynecology & Reproductive Sciences, Yale School of Medicine, Yale University, New Haven, CT 06510, USA; 8 Tri-Institutional Biology and Medicine Program, Weill Cornell Medicine, New York, NY 10021, USA; 9 The Feil Family Brain and Mind Research Institute, Weill Cornell Medicine, New York, NY 10021, USA; 10 WorldQuant Initiative for Quantitative Prediction, Weill Cornell Medicine, New York, NY 10021, USA

**Keywords:** proteomics, genomics, physical activity, spaceflight, scuba diving, airplane flight, simulation racing, multi-omics, resilience, biomarkers, adaptations, human performance, stress response, physiological adaptations

## Abstract

Human engagement in extreme activities, from spaceflight to deep-sea diving and extreme sports, presents unique physiological challenges. Understanding the molecular mechanisms underlying adaptations to these demands is crucial for developing strategies to enhance human performance and resilience in such environments. This review integrates multi-omics data across a range of extreme phenotypes, including astronauts, scuba divers, acute alcohol consumers, long-haul flight passengers, bodybuilders, and simulation racers. We analyze current literature in genomic, transcriptomic, proteomic, metabolomic, and metagenomic studies to identify common and phenotype-specific adaptations, highlighting potential biomarkers and pathways associated with resilience in harsh conditions. This integrated approach offers insights into human adaptability and provides a foundation for developing personalized strategies to mitigate risks and enhance performance in extreme environments, with particular relevance to extended spaceflight.

## 1. Introduction

Human exploration of extreme environments, from the vastness of space to the depths of the ocean and the extremes of physical exertion, presents unparalleled physiological challenges. Extreme environments are habitats or conditions that pose significant physical, chemical, or biological challenges to life, often pushing the limits of survival and adaptation. These environments can be external, such as those encountered during polar missions, space exploration, or deep-sea activities (i.e., scuba diving, freediving) [1,2,3,4,5,6,7,8,9,10,11,12,13,14,15,16,17,18,19,20,21,22,23,24,25,26,27,28,29,30,31,32,33,34,35,36,37,38,39,40,41,42,43,44,45,46,47,48,49,50,51,52,53,54,55,56,57,58,59,60,61,62,63]. Yet, they can also be internal, where the body is exposed to extreme physiological stressors. For example, the toxic effects of alcohol consumption can trigger oxidative stress, inflammation, and systemic metabolic disruption [64,65,66,67,68,69,70,71,72,73,74,75,76,77]. Understanding the molecular mechanisms underlying human adaptation to these harsh conditions is crucial not only for mitigating risks and enhancing performance in these specific activities, but also for gaining broader insights into human resilience and adaptability [1,2,3].

Omics technologies, which encompasses genomics, transcriptomics, proteomics, metabolomics, and metagenomics, provide a powerful toolkit for dissecting these complex adaptations at a molecular level. By integrating data across these different omics layers, we can gain a holistic understanding of the interplay between genes, proteins, metabolites, and the microbiome in response to extreme stressors [1,3,4,5,6,78,79,80,81,82,83,84,85,86]. This integrated, multi-omics approach offers a unique opportunity to uncover novel biomarkers and pathways associated with resilience, paving the way for personalized strategies to optimize human health and performance in extreme environments.

This review integrates and compares findings across a diverse range of extreme phenotypes, each representing a unique set of physiological challenges, aiming to uncover shared adaptive mechanisms and pathways, as well as phenotype-specific responses. By synthesizing this knowledge, we aim to provide a foundation for developing targeted interventions and personalized strategies to mitigate risks and optimize human performance and resilience in extreme environments, with particular relevance to long-duration spaceflight and other challenging scenarios.

## 2. Extreme Phenotypes

This section details known and investigated responses to various physiological stressors (Extreme Phenotypes), as well as gaps for future research. 

### 2.1. Astronauts

Spaceflight exposes individuals to a wide variety of stressors, including microgravity, radiation, confinement, general physiological and psychological stress, confinement, altered day/night cycle exposure, and viral reactivation [1]. Although organisms have evolved on Earth for billions of years, the spaceflight environment is one that is unique for these organisms, and the resultant adaptations reflect this unusual state.

*Genomics:* The SOMA (Space Omics and Medical Atlas) project [2] represents a substantial leap forward in our understanding of astronaut physiological adaptations. Analysis of genomic data from various missions, including the NASA Twins Study [1] and Inspiration4 [3], has revealed a variety of consistent changes. These include cytokine shifts, which indicate an inflammatory response to the stress of spaceflight and immune system alterations [4,5]. While inflammation is a normal response to stress, chronic and excessive inflammation can be detrimental. Telomere elongation has also been found in comparatively large cohorts of astronauts, which is a counterintuitive finding, and whose long term implications are still under investigation [6]. However, this may reflect changes in cellular turnover, DNA repair mechanisms, or microgravity. Alterations in gene expression related to immune function [1,2,3,4,5], DNA damage response [6], and oxidative stress [7] have also been found in multiple studies; these reflect on the viral reactivation noted in astronauts [8], exposure to radiation [9] and (physical, psychological, and environmental) stress astronauts experience. Many changes on a similar scale also occur pre-flight, potentially induced by extensive physical and mental training in a high-stress environment [1,2,3,10].

*Transcriptomics:* Spatially resolved transcriptomics on skin biopsies and cell-free RNA (cfRNA) profiling have revealed novel insights into tissue-specific responses. Notably, cfRNA profiles suggest systemic physiological shifts and reveal subtle changes in cell type proportions during recovery from spaceflight, including emergence of markers for hematopoietic stem cells. These cfRNA changes indicate which tissues are responding most strongly to the stressors, and tissue-specific expressions in different tissue collections [2,11]. Direct RNA sequencing (dRNA-seq) has illuminated changes in RNA modifications associated with radiation and telomere responses. RNA modifications like N6-methyladenosine (m6A) can influence gene expression and play a role in how the body responds to space radiation and telomere elongation [12].

*Proteomics, Metabolomics, and Microbiome:* Proteomic and metabolomic data are integrated within the SOMA resource, offering a comprehensive view of molecular alterations, which supported pathway enrichment related to immune function (antigen binding, immune activation, inflammation) [3], bone metabolism [13], mitochondrial dysfunction and ROS [1,2,3,11,14], and extracellular matrix remodeling [15]. These studies help further elucidate how the changes in genomic and transcriptomics responses to spaceflight play into the shifts in the proteome [1,7]. Further studies leveraging these datasets are ongoing. Metagenomic analyses, while limited, provide preliminary insights into microbial shifts in the astronaut microbiome, in both the gut and the skin [16,17]. These shifts can occur due to altered diet, stress, radiation, altered hygiene practices, and confinement with others while in preflight training and during spaceflight and can impact digestion, immunity, and overall health [16,17,18].

*Gaps and Future Directions:* While SOMA provides unprecedented access to astronaut omics data, larger sample sizes are needed to increase statistical power and enable more robust analyses. Longitudinal studies and the integration of emerging omics technologies, such as single-cell multi-omics and epigenomics, will be crucial for understanding long-term spaceflight effects. Additionally, increasing open science data access to omics datasets from extreme environments, such as polar, high-radiation terrestrial settings, and other environments can provide valuable comparative insights and accelerate cross-disciplinary discoveries for human adaptation and resilience.

*Phenotype-specific adaptations*: The unique environment of spaceflight leads to highly specific adaptations not seen in other phenotypes. Telomere elongation, possibly linked to the combined effects of radiation and microgravity, is a notable example [6]. Understanding the mechanisms driving this phenomenon could have implications for aging research and the development of interventions to mitigate age-related telomere shortening. Spaceflight-associated neuro-ocular syndrome (SANS) is another phenotype-specific adaptation, highlighting the unique challenges posed by microgravity to the visual and nervous systems [18]. Further research using advanced neuroimaging techniques and multi-omics analyses of ocular tissues could provide insights into the pathogenesis of SANS and inform the development of effective countermeasures.

### 2.2. Scuba Divers

Scuba diving exposes the human body to a unique combination of stressors, including hyperbaric pressure, altered respiratory gas mixtures, cold temperatures, and increased physical exertion [20,21,22,23]. The increased pressure underwater can cause barotrauma, affecting air-filled spaces in the body. Breathing compressed gas at depth increases the partial pressure of nitrogen in the blood, which can result in a state of altered mental function and impaired judgment commonly known as ‘nitrogen narcosis’ [24]. Decompression sickness, the formation of bubbles in the tissues and bloodstream, can cause joint pain, paralysis, and long-term neurological symptoms [25]. Diving also places increased strain on the cardiovascular system. These factors can induce significant physiological changes and adaptations, raising concerns about potential long-term health risks [20]. Omics research is beginning to shed light on the molecular mechanisms underlying these adaptations.

*Genomics:* Genetic risk factors for SCUBA diving are not widely studied, but emerging research and analogies from related areas (e.g., high-pressure, oxygen-related, and decompression stress) suggest several key genetic factors that may influence individual risk during diving activities. While not large-scale GWAS has been completed for SCUBA diving, there have been some studies on the and a positively selected variant in a gene previously associated with cold water tolerance, which may contribute to reduced hypothermia susceptibility in female Haenyeo divers from Korea.

*Transcriptomics:* Changes in the blood transcriptome of experienced divers before and after a series of dives to 18 m [21] identified persistent upregulation of genes involved in apoptosis, inflammation, and innate immunity, even before diving—when compared to matched nondiving controls. Diving further induced acute transcriptional changes, primarily affecting CD8+ T cells, NK cells, neutrophils, monocytes, and macrophages. Magri et al. [22] similarly observed transcriptomic changes related to inflammation and innate immunity in divers with decompression sickness. Sureda et al. [23] focused on neutrophil gene expression, finding upregulation of genes related to inflammation and nitric oxide synthesis after a 50 m dive.

*Proteomics:* A recent study by Zarak et al. [20] investigated divers performing weekly dives at 20–30 m depth for 5 weeks. They observed transient increases in protein biomarkers related to cardiac damage (NT-proBNP, hs-Tnl, CK-MB), muscle damage (Mb, galectin-3, CK, LDH), along with suggesting an adaptive response of vascular endothelial activation (ET-1, VEGF), and inflammation (Lkc, CRP, IL-6) in response to damage to the cardiovascular, muscular, and immune systems [20]. Further genomic studies are needed to identify genetic variants influencing susceptibility to diving-related health risks. Lautridou et al. [24] studied the plasma proteome of divers, finding upregulation of complement system proteins after diving, suggesting a role for this system in response to hyperbaric stress. Protein tau concentration has also been shown to increase after scuba diving [26], suggesting neurodegeneration from the stresses of diving. However, other studies have also shown minimal significant differences in the plasma proteome of experienced scuba divers after a single dive [24], showing that adaptation may allow experienced divers to overcome these stresses.

*Metabolomics:* Ciborowski et al. [27] used LC-MS to analyze diver plasma after simulated dives, identifying significant alterations in over 100 metabolites. This included increases in lysophospholipids including lysoplasmalogen, a thrombosis promoter; acylcarnitines; and hemolysis-associated compounds. This study also noted statistically significant changes in metabolites associated with bone degradation, a possible result of the altered resultant gravity that buoyant force from the water provides during scuba diving and water pressure. Perovic et al. [28] observed increased erythrocyte oxidative stress markers immediately after diving and changes in antioxidant enzyme activity in PBMCs.

*Metagenomics:* Monnoyer et al. [29,30] investigated changes in the oral microbiota of divers during a 4-week saturation dive. They found reduced bacterial diversity and shifts in bacterial composition, particularly among obligate anaerobes, and predicted corresponding changes in metabolic pathways related to energy metabolism and oxidative stress. These changes are specifically related to the survival and growth of bacteria in oxygenated environments, namely the increase in aerobic metabolic pathways and decrease in anaerobic pathways.

*Gaps and Future Directions:* Most SCUBA diving studies focus on acute responses. Longitudinal studies are crucial to investigate long-term adaptations and potential health risks associated with chronic diving. Integrating multi-omics data would provide a more holistic understanding of the physiological responses to diving. Studies investigating the impact of learning to dive are also needed, as this adds another layer of physiological and psychological stress. Further research should explore the genetic and epigenetic factors influencing individual responses to diving stressors. Expanding research to other tissue types beyond blood and saliva, such as underwater or immediately post-dive collected skin, bone, or metagenomic samples would also be valuable.

*Phenotype-specific adaptations***:** The hyperbaric environment of deep-sea diving triggers specific adaptations related to gas exchange, pressure regulation, and immune function, and notably this was seen in the recent Polaris Dawn mission (similar to Nitrox gas mixtures). The upregulation of complement system proteins, observed in divers after both single and repeated dives, suggests a role for this system in mediating the response to hyperbaric stress. Further research is needed to investigate the long-term effects of repeated diving on complement activation and its potential implications for immune function and inflammatory processes.

### 2.3. Long-Haul Airplane Passengers

We define a long-haul flight as a flight that lasts more than six hours, to concur with definitions commonly used in the analysis of aerospace physiology. Long-haul air travel presents a unique set of physiological challenges, distinct from other extreme activities discussed in this review. (1) The low-humidity environment in airplane cabins contributes to dehydration and dry skin [31]. Passengers have an increased risk of deep vein thrombosis (DVT) due to prolonged immobility, stasis, and a pro-coagulant state induced by hypoxia [32,33]. (2) Studies have shown increased activation of clotting factors and elevated levels of D-dimer in individuals exposed to high altitudes [34]. (3) Crossing multiple time zones disrupts circadian rhythms, leading to jet lag, characterized by fatigue, irritability, sleep disturbances, and impaired cognitive and physical performance [35,36]. (4) The lower oxygen partial pressure in airplane cabins can also lead to mild hypoxia, potentially affecting various physiological processes. Studies have documented reduced oxygen saturation in passengers during flight [37]. Related to air pressure, changes in air pressure during flight can cause ear pain, perforation, vertigo, and hearing loss due to barotitis, an inflammation of the eardrum [38]. (5) The enclosed environment of an airplane cabin also increases exposure to microbes and can facilitate the spread of infectious diseases [39]. While omics-based investigations into the effects of long-haul flight are currently limited, existing research, primarily focused on physiological changes and preliminary biomarker studies, highlights potential areas for future multi-omics investigations.

*Genomics and Transcriptomics:* Limited genomic and transcriptomic studies have focused on the effects of long-haul flight on DNA damage and gene expression. Minoretti et al. [40] explored the possibility of increased peripheral blood DNA damage and elevated serum MIA (melanoma inhibitory activity) protein in airline pilots, potentially linked to increased skin cancer risk. Further research in this area is warranted to understand the genomic instability associated with long-haul flight and explore potential transcriptional changes related to stress response, immune function, and circadian rhythm disruption. Screening of SERT and p11 mRNA levels, associated with stress and depression, have also shown to be elevated in airline pilots [41]. Findings related to transcriptomics can be mapped against known signals with previously demonstrated relationships to human disease (e.g., Human Cell Atlas [42], HuBMAP [43]).

*Proteomics and Metabolomics:* The proteomic and metabolomic consequences of long-haul flight are largely unexplored. While no specific studies have directly examined these omics profiles in passengers, studies in airline pilots suggest that plasma neurotrophin levels, involved in neuronal and cognitive function, may be affected by occupational factors associated with long-haul flight [40]. Further research is needed to assess potential changes in protein expression and metabolite levels related to inflammation, oxidative stress, and metabolic dysregulation in long-haul flight passengers.

*Metagenomics:* No studies have directly investigated the impact of long-haul flight on the gut microbiome. Given the known effects of the environment, stress, diet, and altered circadian rhythms on gut microbiota composition and function, metagenomic analysis could provide insights into potential dysbiosis and related health consequences associated with prolonged air travel.

*Gaps and Future Directions:* Omics-based research on long-haul airplane passengers is currently in its infancy, highlighting the need for larger-scale studies employing multi-omics approaches to comprehensively assess the molecular changes associated with prolonged air travel. Future research should prioritize longitudinal studies that track omics profiles before, during, and after long-haul flights to capture both acute and long-term adaptations. Concurrently, the integration of multi-omics data, encompassing genomics, transcriptomics, proteomics, metabolomics, and metagenomics, is essential for understanding the complex interplay between different molecular modalities of biology. Investigating individual variability in responses to long-haul flights through personalized medicine, considering factors such as age, sex, pre-existing health conditions, and genetic background, will also be crucial. Furthermore, targeted biomarker discovery is needed to identify reliable indicators for predicting susceptibility to adverse health outcomes like DVT, jet lag, and immune dysfunction. The development and testing of intervention strategies, such as nutritional plans, exercise programs, and light therapy, should aim to mitigate these negative effects.

By addressing these research gaps, we can gain a deeper understanding of the physiological and molecular adaptations to long-haul air travel, paving the way for personalized strategies to enhance passenger health and well-being and reduce thrombosis risk. These insights can also inform strategies for mitigating the negative impacts of prolonged confinement and disrupted circadian rhythms, relevant to other extreme environments, including spaceflight.

*Phenotype-specific adaptations***:** Prolonged immobility and hypoxia contribute to an increased risk of DVT, a specific concern in long-haul flight passengers. Understanding the molecular mechanisms underlying this increased risk, including alterations in blood coagulation pathways and endothelial function, is crucial for developing effective preventative measures. Further research should investigate the potential for combined stressors, including circadian rhythm disruption and dehydration, to exacerbate the risk of DVT.

### 2.4. Bodybuilders

Bodybuilding represents a human phenotype characterized by extreme muscle hypertrophy achieved through a rigorous combination of resistance training, hormesis, and precisely planned nutrition. This unique phenotype offers a valuable model for investigating the complex interplay of genetics, exercise, and nutrition in promoting muscle growth and adaptation. While most omics studies in this area have focused on the effects of short-term resistance training or comparisons between trained and untrained individuals, rather than focusing specifically on competitive bodybuilders, they nonetheless provide a foundation for understanding the molecular mechanisms underlying this extreme phenotype.

*Genomics and Muscle Growth:* Genetic variability plays a significant role in skeletal muscle traits such as strength and mass. Specific genes and gene variants have been identified that contribute to these traits. For example, variations in MSTN (myostatin), a negative regulator of muscle growth, has been linked to muscle strength and hypertrophy. Loss-of-function mutations in MSTN result in significant muscle growth. Other genes, such as CDKN1A, MYOD1, and ACVR1B, also play crucial roles in muscle growth and differentiation pathways, including the myostatin signaling pathway [44]. Research using transgenic mouse models has further identified genes like Ski, Ppargc1a, and Yap1, involved in muscle protein synthesis, mitochondrial function, and cell signaling, whose manipulation can influence muscle mass [45]. A more recent study by Venckunas et al. [46] found associations between nine specific SNPs in muscle-growth-related genes and muscle mass and strength in young men, suggesting a genetic predisposition to training adaptations.

*Transcriptomic Responses to Resistance Training:* Transcriptomic studies have explored how resistance exercise alters gene expression in skeletal muscle. Raue et al. [47] analyzed gene expression changes in muscle biopsies from trained and untrained men after a single bout of resistance exercise, observing distinct transcriptomic responses between the two groups, indicating that prior training influences acute exercise-induced gene expression. Russell et al. [48] profiled microRNA expression, identifying specific microRNAs differentially expressed between strength-trained athletes and sedentary controls, suggesting a role for these molecules in regulating muscle growth and adaptation. Thalacker-Mercer et al. [49] conducted a genomic microarray analysis and found that individuals who responded most strongly to resistance training exhibited distinct gene expression patterns related to skeletal muscle development and reduced proinflammatory signaling, suggesting a possible “primed” state for muscle growth.

*Proteomic Adaptations:* Proteomic analyses have shed light on the complex protein-level changes that occur in response to resistance training. Schönke et al. [50] examined protein expression changes in various muscle types, finding that proteins related to transcription, mitochondrial metabolism, calcium signaling, and nutrient metabolism adapt to training in a fiber type-specific manner. Vann et al. [51] compared strength and proteomic profiles in athletes, observing distinct strength increases but no significant changes in sarcoplasmic protein concentrations or signaling and metabolic pathways. Other studies highlight the importance of training volume and load in influencing proteomic adaptations [51,52,53].

*Metabolomic and Metagenomic Considerations:* The extreme dietary practices of bodybuilders, characterized by cyclical periods of bulking and cutting, have important metabolic and metagenomic implications. Schranner et al. [54] found distinct blood metabolite profiles in natural bodybuilders compared to other athletes and untrained individuals, including differences in phosphatidylcholines and branched-chain amino acids. Parstorfer et al. [55] identified further alterations in lipid and carbohydrate metabolism in strength-trained athletes. Metagenomic studies have revealed unique gut microbial compositions in bodybuilders [56,57], with potential implications for muscle growth and adaptation due to reduced abundance of short-chain fatty acid producers [58,59]. The gut-muscle axis, influenced by both exercise and diet, is a crucial area for future investigation in this population.

*Gaps and Future Directions:* Research on the bodybuilder phenotype is still in its early stages. Several key gaps need to be addressed. Firstly, most existing research examines trained individuals rather than competitive bodybuilders. Studies focusing specifically on this extreme phenotype, with their unique training and dietary practices, are needed to assess the effects of such a lifestyle and associated dietary, circadian, and metabolic patterns. The long-term impact of cyclical high-protein and calorie-restricted diets on overall health, metabolic adaptation, and the gut microbiome also require further investigation. Understanding the role of individual genetic predispositions, training regimens, and dietary choices in influencing responses to bodybuilding practices is crucial. Combining data across genomic, transcriptomic, proteomic, metabolomic, and metagenomic platforms would provide a more holistic understanding of the complex interplay between these factors in driving muscle hypertrophy and recovery. Long-term studies are needed to assess the potential health consequences of extreme bodybuilding practices, including the impact on cardiovascular, renal, and hepatic function.

By addressing these gaps, we can gain a more complete understanding of the molecular mechanisms underlying muscle hypertrophy in bodybuilders, with implications for developing targeted interventions to maximize muscle growth while mitigating potential health risks.

*Phenotype-specific adaptations***:** The extreme muscle hypertrophy observed in bodybuilders is a phenotype-specific adaptation driven by the interplay of resistance training and specialized nutrition. The shifts in gut microbiome composition, characterized by a reduced abundance of short-chain fatty acid producers, may represent a novel adaptation to high-protein diets, but its long-term consequences for gut health and metabolic function require further investigation.

### 2.5. Simulation Racers

Competitive simulator racing, particularly in the realm of motorsport, has rapidly gained popularity, providing a unique platform for both amateur and professional drivers to hone their skills and compete in virtual environments. While real-world motorsport has been the prior subject of physiological studies, the specific demands and potential health implications of competitive simulator racing remain largely unexplored from a multi-omics or physiological perspective. This represents a significant gap in our understanding of human adaptation to extreme performance in virtual environments.

*Physiological Demands and Cognitive Characteristics:* Simulator racing, while lacking the physical G-forces, vibrations, and extreme temperatures of real-world motorsport, still presents unique physiological and cognitive demands. Studies have shown that simulation racers, like real-world drivers, exhibit distinct cognitive patterns and neurological utilization compared to controls [60]. These characteristics include enhanced perceptual speed, improved motor skills, and specific oculomotor behaviors, such as pairing gaze direction with head movement, suggesting a high degree of hand-eye coordination. Furthermore, studies using brain MRI have revealed differences in brain activation patterns between racing drivers and controls, with racing drivers showing greater neural efficiency and stronger functional connections in areas related to motor control and spatial navigation [60]. While these findings are primarily derived from real-world drivers, it is plausible that similar cognitive and neurophysiological adaptations may also exist among competitive simulation racers.

Beyond cognitive demands, competitive simulator racing also involves sustained periods of intense focus and concentration, often lasting for several hours. This prolonged mental exertion can lead to fatigue and stress, which may have both acute and long-term health consequences. The physical demands, while less intense than real-world racing, still involve sustained muscle activation for controlling the steering wheel, pedals, and other input devices. This can result in localized muscle fatigue, particularly in the hands, arms, and shoulders, as well as potential for repetitive strain injuries.

*Existing Physiological Studies and Limitations:* A handful of studies have examined the physiological responses to simulated driving, but these primarily focus on general driving tasks rather than competitive racing scenarios. Georgiou et al. [61] investigated basic physiological indicators like heart rate, eye gaze, and head movement in simulator drivers, but did not find significant differences in heart rate between high-level performers and controls. Johnson et al. [62] conducted a more comprehensive physiological analysis, including heart rate, ventilation, and oxygen consumption, and observed significant changes in these parameters during simulated driving compared to baseline, but these changes were evoked by surprising events rather than representative of competitive racing conditions. Filho et al. [63] measured psychophysiological responses in professional racing drivers during simulator sessions, noting inter-individual differences in profiles, but lacked a control group for comparison. These studies, while providing initial insights, highlight the need for further research specifically focused on the physiological demands of competitive simulator racing.

*Multi-Omics Profiling:* Despite the growing popularity of competitive simulator racing, no known studies have investigated the associated biomarkers using a multi-omics or modern molecular approach. This represents a significant opportunity to delve deeper into the molecular mechanisms underlying adaptation to this unique activity, and also to compare to race car drivers. Such work could identify changes in gene and protein expression, metabolite levels, and gut microbiome composition related to the prolonged mental and physical exertion of competitive simulator racing. Long-term, these methods could also detail the impact on metabolic and immune functions, by assessing the effects of prolonged sitting, altered sleep patterns, and dietary habits often associated with competitive gaming on metabolic and immune health.

*Future Research Directions:* To fully elucidate the physiological and molecular adaptations associated with competitive simulator racing, future research should prioritize standardized protocols, which will ensure comparability across studies, facilitate meta-analyses, and allow integration with performance data. Correlating omics data with objective and race-relevant performance metrics, such as lap times, consistency, and input telemetry, will allow for the identification of potential biomarkers of elite performance. Longitudinal monitoring and gene-set and pathway analyses will allow for the identification of changes in profiles over time (to capture acute and long-term adaptations) as well as gene set and pathway analysis to allow for the identification of pathways associated with this stress and enhanced performance. This framework will allow for comprehensive studies incorporating genomic, transcriptomic, proteomic, metabolomic, and metagenomic analyses in sim-racers compared to appropriate control groups.

By addressing these research gaps, we can gain valuable insights into the physiological and molecular underpinnings of performance in virtual motorsport, with potential implications for optimizing training strategies, mitigating health risks, and understanding the broader implications of prolonged engagement in virtual environments. Furthermore, the unique characteristics of simulator racing, such as its controlled environment and accessibility, make it a valuable model for studying the broader aspects of human adaptation to extreme performance and stress in the digital age.

*Phenotype-specific adaptations***:** While sharing some characteristics with real-world racing drivers, simulation racers also present unique adaptations related to prolonged engagement in virtual environments. Further research using multi-omics profiling is needed to fully characterize the physiological and neurological demands of competitive simulator racing, including the impact on stress response pathways, metabolic function, and the gut microbiome.

### 2.6. Acute Alcohol Consumption

Acute alcohol consumption, defined as consuming a large amount of alcohol in a short period leading to a blood alcohol concentration (BAC) of 0.08 g/dL or higher, is a prevalent public health concern. Even in individuals without alcohol use disorder, acute alcohol exposure can induce significant physiological and molecular changes, impacting various organ systems and pathways. Omics research offers valuable insights into the mechanisms underlying these changes and their potential long-term consequences.

*Genomic and Epigenetic Influences:* Genetic factors play a crucial role in alcohol metabolism and individual susceptibility to alcohol dependence. Variants in genes encoding alcohol dehydrogenase (ADH) and aldehyde dehydrogenase (ALDH), the primary enzymes responsible for alcohol metabolism, influence how individuals respond to alcohol. For instance, specific ALDH2 variants (the ALDH2*2 variant) can lead to unpleasant effects after alcohol consumption through a buildup of acetaldehyde causing symptoms like facial flushing, nausea, and heart palpitations. This potentially reduces the risk of developing alcohol use disorder [64]. Genome-wide association studies (GWAS) have identified numerous genetic loci associated with alcohol dependence and related traits, implicating genes involved in neurotransmitter signaling, stress response, and reward pathways [65]. Furthermore, even short-term drinking (defined by an absence of liver fibrosis markers and transient gene expression changes as opposed to persistent ones) can induce epigenetic modifications, including DNA methylation and histone modifications, affecting gene expression and potentially contributing to long-term consequences like alcohol dependence [66].

*Transcriptomic Responses in Brain and Liver:* Alcohol consumption significantly alters gene expression in various tissues, particularly the brain and liver. Although animal models cannot be completely relied on here—as they have specific physiology and organ-specific traits in extreme phenotypes—acute alcohol exposure has been shown to induce changes in gene expression related to neuroinflammation, synaptic plasticity, and stress response pathways in the brain [67,68,69]. In the liver, alcohol consumption affects gene expression related to lipid metabolism, inflammation, and oxidative stress [70]. Alcohol also modulates the expression of genes involved in immune function, potentially contributing to its immunosuppressive effects [71].

*Proteomic and Post-Translational Modifications:* Proteomic analyses have identified alterations in protein expression and post-translational modifications following alcohol consumption. In the brain, changes have been observed in proteins involved in neurotransmitter signaling, cytoskeletal structure, and oxidative stress response [72]. In the liver, alcohol consumption impacts protein expression related to detoxification, lipid metabolism, and inflammation [73].

*Metabolomic Shifts:* Metabolomic profiling has revealed alterations in various metabolites after alcohol consumption. In the liver, changes occur in metabolites related to lipid metabolism, amino acid metabolism, and oxidative stress [74]. Similarly, alterations in brain metabolites associated with neurotransmitter and energy metabolism have been observed in animal models after acute alcohol exposure [75].

*Metagenomic Disruptions:* Alcohol consumption can also disrupt the gut microbiome composition and function. Acute alcohol exposure can lead to gut microbiota dysbiosis, characterized by changes in the abundance and diversity of bacterial species [76]. This dysbiosis can contribute to gut inflammation and increased intestinal permeability. Furthermore, alterations in the gut microbiome can impact the production of microbial metabolites, such as short-chain fatty acids (SCFAs), which play important roles in host metabolism and immunity [77].

*Gaps and Future Directions:* While omics approaches have advanced our understanding of the molecular consequences of acute alcohol consumption, several research gaps remain. Firstly, more human studies are needed, particularly longitudinal studies that track omics changes over time (longitudinal analyses) to assess both the acute and long-term effects of acute alcohol consumption. Integration of multi-omics data from different omics platforms is critical to provide a more comprehensive understanding of the interconnected molecular responses to alcohol exposure. Future research should also focus on understanding the individual variability in responses to alcohol, considering factors such as genetic background, age, sex, medications, and pre-existing health conditions; such a framework is crucial for developing personalized preventative and therapeutic interventions. Lastly, further exploration on the impact of alcohol on traditionally unrelated tissues, such as the heart and lungs, would be beneficial for more targeted therapeutic interventions.

By addressing these gaps, we can gain a more complete picture of the molecular underpinnings of drinking alcohol and its impact on human health, ultimately informing the development of more effective strategies for prevention and treatment.

*Phenotype-specific adaptations:* The toxic effects of acute alcohol exposure lead to distinct molecular changes, particularly in the liver and brain. Alterations in gene and protein expression related to detoxification pathways, inflammation, and oxidative stress are characteristic of this phenotype. Further research is needed to understand the individual variability in responses to alcohol consumption and to identify biomarkers that can predict susceptibility to alcohol-related liver and brain damage. A summary of this section is presented in Table 1. 

## 3. Cross-Phenotype Analysis and Integrated Insights: Shared Adaptations and Unique Challenges

The diverse array of extreme phenotypes examined in this review, while each presenting unique physiological challenges, also reveals shared adaptive mechanisms and common molecular pathways activated in response to extreme stressors. Integrating insights across these different phenotypes can provide a more holistic understanding of human resilience and adaptability, and offers potential for leveraging knowledge from one phenotype to inform research and intervention strategies for another.

**Shared Adaptations:**

A recurring theme across these various stressors is the activation of generalized stress response pathways. However, a deeper molecular analysis reveals that while the cellular “alarm systems” are conserved, the specific pathways engaged are fine-tuned to the nature of the threat. Across multiple phenotypes—astronauts, scuba divers, alcohol consumers, and long-haul flight passengers—we observe consistent upregulation of genes and proteins related to inflammation [1,3,4,5,20,21,24,27,28,45,73], oxidative stress [1,3,7,20,21,22,23,24,28,32,33,45,50,66,70,72,74,75], and DNA damage response [6,26,40,41,64,66]. This points to a conserved, fundamental mechanism for dealing with cellular stress induced by a diverse range of extreme or stressful conditions.

Comparing the molecular triggers and downstream cascades reveal important parts of this mechanism. For instance, the stress response in astronauts is heavily influenced by space radiation, which induces DNA double-strand breaks and activates the DNA damage response (DDR) pathway, with key roles for signaling molecules like ATM and p53. In contrast, the primary stressor for a scuba diver is often hyperoxia, which generates a surge of reactive oxygen species (ROS). This predominantly activates antioxidant defense pathways, such as the Nrf2 signaling cascade, leading to the upregulation of enzymes like superoxide dismutase and catalase to neutralize oxidative damage. While both scenarios may result in a general inflammatory state, often mediated by the NF-κB pathway, the initial triggers are fundamentally different. Similarly, while the upregulation of heat shock proteins (HSPs) is a common feature indicating cellular stress [78], their induction in an astronaut [1,3,4] might be a response to radiation-induced protein misfolding, whereas in a bodybuilder, athlete [79], or individual undergoing psychological stress [80] it is likely a response to exercise-induced cellular strain. Therefore, it is more accurate to view this not as a single “generalized response,” but as a shared toolkit of molecular modules that are deployed with different priorities and kinetics depending on the specific environmental challenge. Further investigation into the common regulatory networks governing these shared stress responses could reveal key targets for enhancing resilience across a spectrum of extreme environments.

Extreme activities frequently necessitate significant shifts in energy metabolism. We see evidence of metabolic reprogramming across multiple phenotypes, particularly those involving intense physical exertion. Both bodybuilders and marathon runners demonstrate substantial alterations in lipid and carbohydrate metabolism [81,82], mitochondrial function [83,84], and amino acid oxidation [82,85,86]. This involves the upregulation of pathways for glucose uptake and glycolysis to provide immediate ATP and the diversion of metabolites into anabolic pathways like the pentose phosphate pathway and nucleotide synthesis to support tissue repair and hypertrophy, often driven by the Pi3k-Akt-mTOR signaling axis. These changes reflect the increased energy demands and the need for efficient fuel utilization during and after periods of extreme physical stress.

However, the nature of the stressor dictates unique metabolic signatures. In the microgravity environment of spaceflight, for instance, omics data has shown an upregulation of hepatic lipid metabolism, including fatty acid biosynthesis, yet a concurrent downregulation of mitochondrial oxidative phosphorylation and insulin signaling pathways in muscle tissue, potentially contributing to the documented development of insulin resistance. In contrast, the hyperbaric and hyperoxic stress of SCUBA diving triggers a different adaptive response. Metagenomic analysis of divers’ oral microbiota reveals a distinct shift from anaerobic to aerobic metabolic pathways to capitalize on increased oxygen availability, while proteomic studies suggest an upregulation of endogenous antioxidant systems to counteract oxidative stress. These divergent strategies; anabolic redirection in bodybuilders, lipid dysregulation in astronauts, and oxidative adaptation in divers; highlight how different extreme conditions co-opt and modulate core metabolic networks. A deeper comparative analysis of these metabolic adaptations could reveal common strategies for optimizing energy production and utilization under extreme conditions. Exploring the interplay between these metabolic shifts and the gut microbiome, which also undergoes significant changes in several phenotypes, is another promising avenue for future research.

The disruption of circadian rhythms and sleep patterns is another common challenge in many extreme activities, including spaceflight and long-haul air travel. However, while the core issue is shared, the nature and magnitude of the molecular dysregulation differ significantly based on the stressor. Both scenarios disrupt the body’s central circadian pacemaker, the suprachiasmatic nucleus (SCN), leading to a stark reduction in the number of rhythmic transcripts that govern physiological homeostasis. Core targets here include clock genes like CLOCK and ARNTL (BMAL1) as well as the machinery for transcription and translation.

The primary distinction lies in the severity and chronicity of the disruption. Long-haul flights induce an acute misalignment, where the extent of pathway dysregulation is generally proportional to the number of time zones crossed, with the body eventually re-entraining to the new light-dark cycle. In contrast, the spaceflight environment presents a more profound and continuous challenge. Astronauts in low Earth orbit experience a sunrise and sunset approximately every 90 min, which can desynchronize circadian rhythms between different tissues. Studies in animal models have revealed that spaceflight can alter the rhythmic amplitude of core clock genes like PER2 in peripheral organs such as the liver, independent of the central clock in the SCN. This suggests that while long-haul travel causes a systemic phase shift, spaceflight may induce a more chaotic, internal desynchrony among organ systems. Further investigation into the transcriptomic changes in pathways governing glucocorticoid signaling and metabolic function across these phenotypes could provide valuable insights into the shared and unique effects of these stressors on circadian regulation.

## 4. Integrating Insights for Translational Applications and Spaceflight

The comparative analysis of extreme phenotypes provides a unique opportunity to leverage knowledge from one area to inform research, countermeasures, and interventions in another. For example, the insights gained from studying muscle wasting in astronauts could potentially be applied to developing interventions for sarcopenia, an age-related decline in muscle mass and function. Similarly, the research on circadian rhythm disruption in long-haul flight passengers and shift workers could inform the development of countermeasures for astronauts experiencing sleep disturbances in space. Specifically, regarding extended spaceflight, integrating knowledge gained from these diverse phenotypes offers three crucial advantages:

(1) **Shared Stress Adaptation Mechanisms**: Understanding shared adaptations, such as the generalized stress response and metabolic reprogramming, can inform the development of countermeasures applicable to multiple stressors encountered during spaceflight. For instance, targeted nutritional interventions or pharmacological agents that modulate inflammation and oxidative stress could benefit astronauts coping with the combined effects of radiation, microgravity, and confinement.
(2) **Personalized Risk Stratification and Countermeasures**: As with terrestrial extreme activities, individual variability in physiological and molecular responses is also evident amongst astronauts, highlighted by differences in their susceptibility to spaceflight-related health risks. Moving beyond population averages to a truly personalized risk assessment requires a sophisticated framework for integrating multi-omics data across platforms. The process begins with data harmonization, using standardized protocols and ontologies to ensure that genomic, transcriptomic, proteomic, and metabolomic datasets are comparable. Then, integrative analytical tools, such as machine learning algorithms and network-based modeling, can identify predictive signatures by correlating, for example, specific genetic variants with downstream changes in protein expression and metabolite levels under stress. Rather than relying on a single biomarker, this integrated method allows for the creation of multi-dimensional, composite risk scores. For example, an astronaut’s susceptibility to spaceflight-associated neuro-ocular syndrome (SANS) might be predicted by a score that combines genetic predispositions for fluid shifts, transcriptomic markers of inflammation in the blood, and metabolomic indicators of oxidative stress. These omics profiles are additionally not static and can be monitored longitudinally throughout a mission. Tracking an individual’s molecular response over time provides a dynamic assessment of their health status and allows for the real-time adjustment of countermeasures. This multi-omics approach enables a shift from reactive to predictive health management, allowing for the development of personalized countermeasures tailored to an individual astronaut’s unique biological response to the spaceflight environment. Integrating omics profiling with physiological monitoring could enable the development of personalized countermeasures tailored to individual astronaut needs.
(3) **Longitudinal Models for Health Risk Prediction**: Longitudinal omics studies in other extreme phenotypes, such as long-haul pilots or deep-sea divers, can provide valuable insights into the potential long-term health consequences of exposure to stressors relevant to spaceflight, such as radiation, hypoxia, and circadian rhythm disruption.

## 5. Future Directions for Integrated Research

Developing standardized protocols and data analysis pipelines to facilitate the integration of multi-omics data across different extreme phenotypes is crucial. This will require collaborative efforts to harmonize data formats, ontologies, and analysis methods. Additionally, novel sample collection processes, leveraging strategies such as blood microsampling, during extreme activities should be explored, to obtain specimens that can generate multi-omics data reflective of biological changes during the activity. Understanding the sex differences in responses to extreme environments and activities is also crucial, and still emerging across all these fields.

Applying systems biology approaches and computational modeling can help us decipher the complex interplay between different molecular layers and pathways involved in adaptation to extreme environments. This could lead to the identification of key regulatory networks and potential therapeutic targets. Understanding these molecular signatures will allow us to uncover principles of resilience applicable not only to astronauts and athletes, but also to aging and chronic diseases. Rigorous validation of potential biomarkers identified through omics analyses is essential for translating these findings into clinical practice. This will require large-scale, longitudinal studies and the development of robust assays for measuring these biomarkers.

As omics technologies become more powerful and personalized, it is crucial to consider the ethical implications of using this information, especially in the context of extreme activities with a limited number of participants. Protecting individual privacy, ensuring equitable access to interventions, and mitigating potential risks of genetic discrimination are important considerations. At the same time, promoting open science policies and fostering interdisciplinary collaboration are essential for handling data from extreme environments, as these datasets are often unique to specific conditions and missions and are not easily reproducible. By pursuing these future directions, we can harness the power of multi-omics profiling and cross-phenotype analysis to unlock deeper insights into human adaptability and resilience, paving the way for safer and more successful human exploration of extreme environments, from the depths of the ocean to the vastness of space.

## 6. Conclusions

Multi-omics profiling provides a powerful lens for investigating human adaptation to extreme environments. By integrating data across diverse phenotypes, we have highlighted common adaptive mechanisms, including alterations in immune function, DNA damage response, heat shock, and metabolic pathways. Phenotype-specific adaptations have also been revealed, highlighting the unique challenges posed by different extreme activities. This integrated approach holds immense promise for developing personalized strategies to mitigate risks, enhance resilience, and optimize human performance in harsh conditions. As we continue to push the boundaries of human exploration, a deeper understanding of these adaptive mechanisms will be essential for ensuring the health and safety of individuals venturing into extreme environments, particularly during extended spaceflight missions.

## Figures and Tables

**Table 1:** An overview of key stressors, multi-omic findings, and key phenotype-specific adaptations for various Extreme Phenotypes (Background color added to improve readability).

Phenotype	Key Stressors	Genomic/Epigenetic Findings	Transcriptomic Findings	Proteomic /Metabolomic Findings	Metagenomic Findings	Key Phenotype-Specific Adaptations
Astronauts	Microgravity, radiation, confinement, altered day/night cycle, psychological stress.	Telomere elongation, DNA damage responses, alterations in genes related to immune function and cytokine shifts.	Cell-free RNA (cfRNA) profiles show systemic physiological shifts; direct RNA sequencing reveals m6A modifications linked to radiation/telomere response.	Upregulation of pathways for immune function, bone metabolism, mitochondrial dysfunction, and extracellular matrix remodeling.	Shifts in both gut and skin microbiome composition due to altered diet, stress, and confinement.	Telomere elongation in response to spaceflight; development of Spaceflight-Associated Neuro-Ocular Syndrome (SANS).
Scuba Divers	Hyperbaric pressure, altered respiratory gas mixtures, cold temperatures, physical exertion.	Not widely studied, but some variants are linked to cold water tolerance in specific populations (e.g., Haenyeo divers).	Persistent upregulation of genes for inflammation, apoptosis, and innate immunity. Acute changes affecting T cells, NK cells, and neutrophils.	Transient increases in markers for cardiac/muscle damage (NT-proBNP, CK-MB) and inflammation (CRP, IL-6). Upregulation of complement system proteins.	Reduced diversity and shifts in the oral microbiota, with an increase in aerobic metabolic pathways.	Upregulation of the complement system in response to hyperbaric stress is a notable adaptation.
Long-Haul Airplane Passengers	Prolonged immobility, low humidity, cabin hypoxia, circadian rhythm disruption (jet lag), pressure changes.	Limited studies; potential for increased DNA damage and elevated stress-related mRNA levels (SERT, p11) noted in airline pilots.	Largely unexplored but can be mapped against known human disease atlases.	Largely unexplored; some evidence in pilots suggests effects on plasma neurotrophin levels.	No direct studies available; dysbiosis is hypothesized due to environmental and circadian factors.	Increased risk of Deep Vein Thrombosis (DVT) due to prolonged immobility and a hypoxic, pro-coagulant state.
Bodybuilders	Rigorous resistance training, cyclical high-protein and calorie-restricted diets (bulking/cutting).	Genetic variability in genes regulating muscle growth and mass, such as MSTN (myostatin).	Distinct gene expression patterns in skeletal muscle related to training status and response to exercise; specific microRNAs regulate muscle adaptation.	Alterations in proteins related to mitochondrial metabolism, calcium signaling, and nutrient metabolism; distinct blood metabolite profiles.	Unique gut microbial compositions, characterized by a reduced abundance of short-chain fatty acid producers.	Extreme muscle hypertrophy driven by the interplay of resistance training and nutrition; gut microbiome shifts potentially related to high-protein diets.
Simulation Racers	Prolonged and intense mental concentration, localized muscle fatigue, potential repetitive strain injuries.	No known multi-omics studies have been conducted. This represents a significant research gap.	No known multi-omics studies have been conducted.	No known multi-omics studies have been conducted.	No known multi-omics studies have been conducted.	Neurological adaptations including enhanced perceptual speed, motor skills, and greater neural efficiency in motor control areas.
Acute Alcohol Consumers	Systemic toxicity from alcohol and its metabolites (e.g., acetaldehyde).	Genetic variants in alcohol metabolism genes (ADH, ALDH) influence response; epigenetic modifications (DNA methylation) affect gene expression.	Altered gene expression in the brain (neuroinflammation, synaptic plasticity) and liver (lipid metabolism, inflammation).	Alterations in proteins involved in detoxification, lipid metabolism, neurotransmitter signaling, and oxidative stress response.	Gut microbiome dysbiosis, characterized by changes in bacterial diversity and increased intestinal permeability.	Distinct molecular damage signatures concentrated in the liver and brain, related to detoxification pathways, neurotoxicity, and inflammation.

## Data Availability

No new data were created or analyzed in this study. Data was derived from resources available in the public domain.
